# MICP as a potential sustainable technique to treat or entrap contaminants in the natural environment: A review

**DOI:** 10.1016/j.ese.2021.100096

**Published:** 2021-05-13

**Authors:** Adharsh Rajasekar, Stephen Wilkinson, Charles K.S. Moy

**Affiliations:** a Jiangsu Key Laboratory of Atmospheric Environment Monitoring and Pollution Control (AEMPC), Collaborative Innovation Center of Atmospheric Environment and Equipment Technology (CIC-AEET), Nanjing University of Information Science &Technology, Nanjing, 210044, China; b Department of Civil Engineering, University of Wollongong in Dubai, Dubai, United Arab Emirates; c Department of Civil Engineering, Xi'an Jiaotong Liverpool University, Suzhou, Jiangsu, China

**Keywords:** Biomineralization, MICP, Urease enzyme, Heavy meal entrapment

## Abstract

In the last two decades, developments in the area of biomineralization has yielded promising results making it a potentially environmentally friendly technique for a wide range of applications in engineering and wastewater/heavy metal remediation. Microbially Induced Carbonate Precipitation (MICP) has led to numerous patented applications ranging from novel strains and nutrient sources for the precipitation of biominerals. Studies are being constantly published to optimize the process to become a promising, cost effective, ecofriendly approach when compared with the existing traditional remediation technologies which are implemented to solve multiple contamination/pollution issues. Heavy metal pollution still poses a major threat towards compromising the ecosystem. The removal of heavy metals is of high importance due to their recalcitrance and persistence in the environment. In that perspective, this paper reviews the current and most significant discoveries and applications of MICP towards the conversion of heavy metals into heavy metal carbonates and removal of calcium from contaminated media such as polluted water. It is evident from the literature survey that although heavy metal carbonate research is very effective in removal, is still in its early stages but could serve as a solution if the microorganisms are stimulated directly in the heavy metal environment.

## Highlights

• Microbially induced carbonate precipitation as a sustainable method is reviewed.
• Latest development of MICP towards the treatment of contaminants is reported.
• The role of urease-secreting bacteria (USB) is discussed.
• The application of heavy metal entrapment and calcium removal is discussed.
• The efficiency of heavy metal removal by MICP is reviewed.

## Graphical abstract

**Figure:**
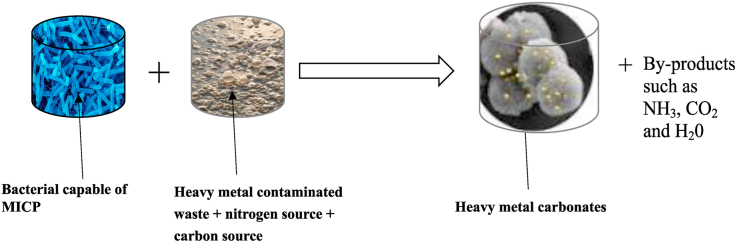

## 1 Introduction

### 1.1 History and concept of biomineralization

Biomineralization is the precipitation of wide range of minerals by living organisms [1]. Organisms belonging to all five kingdoms have been identified to precipitate minerals [2]. Studies of the mineral formation mechanisms, structures and environments, have led to the consideration of how biomineral formation can be directed and applied for human benefit in engineering projects [[3], [4], [5]]. The potential for the biomineralization of heavy metal entrapment has been hypothesized but with few studies showing possible heavy metal entrapment [[6], [7], [8], [9]]. Recently, fungi isolated from soil that have the ability to produce urease enzyme have also been shown to be successful in precipitating metal carbonates [10]. Application of Microbially Induced Carbonate Precipitation (MICP) for heavy metal entrapment results in the production of heavy metal carbonates. In geotechnical engineering, the ultimate goal of utilizing biominerals is to improve the ground in terms of increasing strength and reducing permeability. Biomineralizing technology is considered to have a higher long-term sustainability in comparison to more traditional techniques such as soil washing, composting and thermal incineration [6,7,[11], [12], [13], [14], [15], [16], [17]]. In addition, due to its low energy requirement and ability to retain carbon in the ground, it has attracted attention as a possible carbon negative technique in construction [18,19]. [20] proposed that biomineralization occurs either through biologically-controlled or biologically-induced mineralization processes and sometimes both in combination. Biologically-induced mineralization process is often shown in the literature as the most suitable mechanism for carbonate precipitation [21].

### 1.2 Biomineralization through microbially induced carbonate precipitation (MICP)

In past two decades, MICP has gained a lot of attention in the scientific community leading to multiple publications assessing the different methodologies and outcomes [[22], [23], [24], [25], [26], [27], [28], [29], [30]]. This technique relies on the metabolic activity of the microbes by manipulating the physicochemical conditions of their environment in order to precipitate certain types of biominerals.

Numerous research studies have theorized that carbonate precipitation by bacteria is “induced”, meaning that the type of mineral that's precipitated during the process is highly dependent on the environmental conditions [[31], [32], [33], [34], [35], [36], [37], [38], [39], [40]]. We believe the theory behind “induced” refers to the crystalline nature of precipitated carbonate polymorphs such as calcite, dolomite, vaterite, hydroxyapatite and aragonite being highly dependent on abiotic factors rather than the bacteria or enzymes. Bacteria from diverse environments, as well as abiotic factors (e.g., nutrient composition, salinity), have been found to influence the efficiency and stability of carbonate polymorphs [[41], [42], [43], [44]]. The key factors that contribute to successful and efficient calcium carbonate precipitation is a combination of biological and chemical processes that include: (1) pH, (2) temperature, (3) substrate medium and (4) bioavailability of microorganisms [21,[45], [46], [47]].

**Fig. 1:**
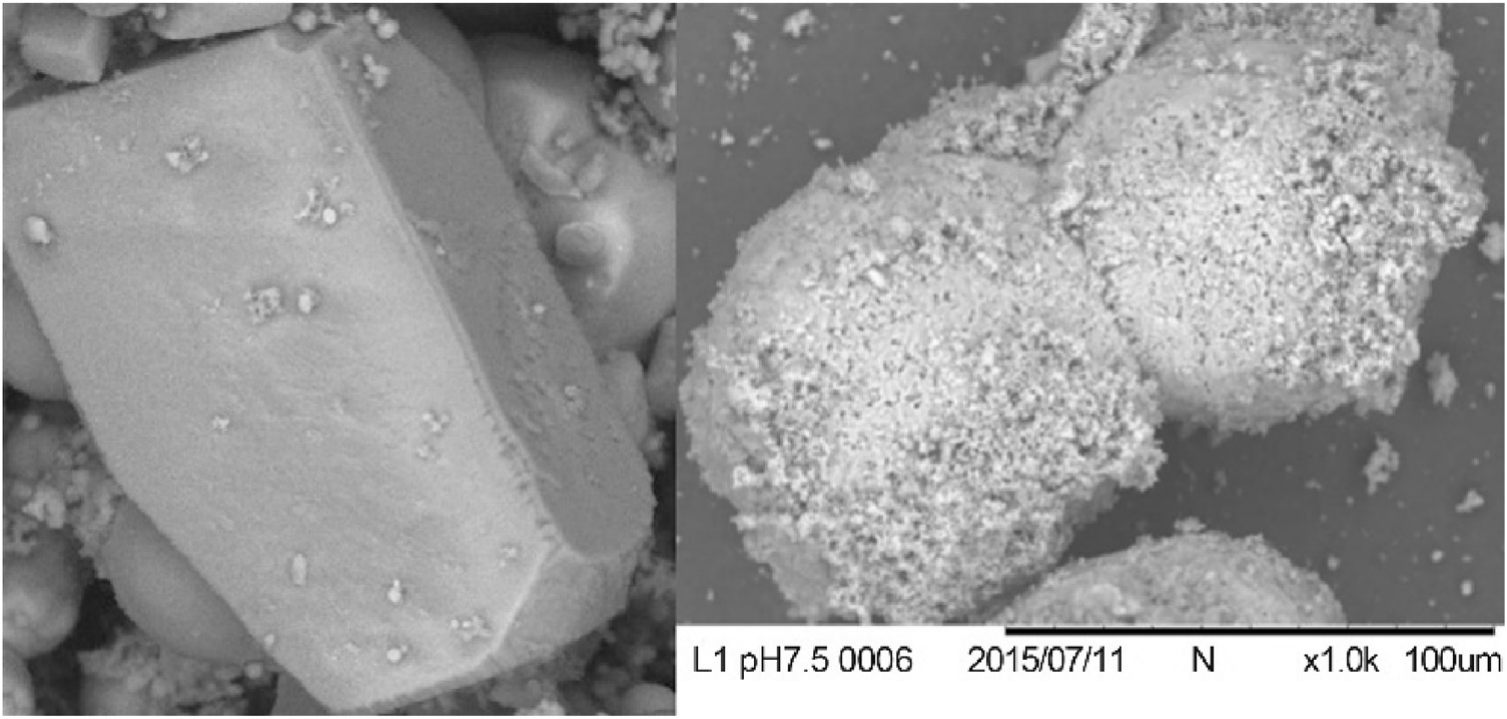
Calcium carbonate polymorphs

Irrespective of these two processes, the biominerals that are precipitated by bacteria are usually in the nano-to micro-size ranges and the composition that results from these processes are usually a mixture of various polymorphs (Fig. 1). This composition is useful in soil engineering as microbes can enter the void spaces and alter the chemical conditions of the pore fluid allowing crystals to form.

## 2 Microbially induced carbonate precipitation (MICP): Urease enzyme

**Fig. 2:**
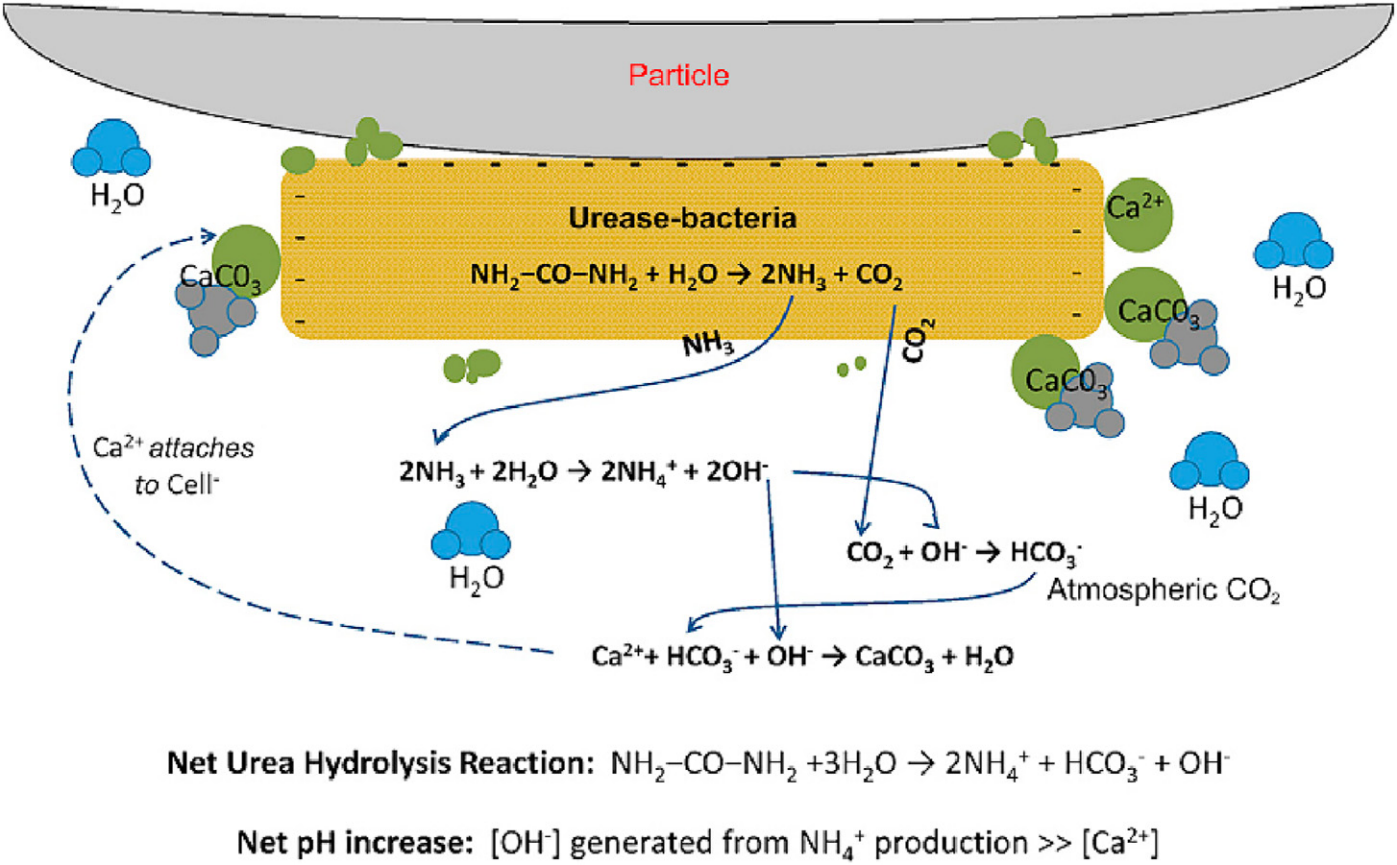
Urea hydrolysis resulting calcium carbonate formation. Modified from (Dhami et al., 2013)

Although several processes, such as photosynthesis, ammonification, denitrification, are associated with and studied for their ability to precipitate carbonate polymorphs, ureolysis or urea hydrolysis is the commonly applied technique when it comes to calcium carbonate precipitation [21]. Several microorganisms have been studied for their ability to precipitate carbonate via ureolysis (Table 1). For an efficient ureolysis, a nitrogen source has to be supplied for carbonate precipitation to occur. Urea is a nitrogen source and also highly cost effective when compared to other nitrogen sources such as ammonium chloride. Urease enzyme activity by microorganisms is sensitive to multiple parameters such as temperature (which is expected to be at 35 °C for superior activity), pH (7–8.5 for superior activity), nitrogen source and incubation period [22,26,48]. One of the most studied urease enzyme secreting bacteria (USB) used for MICP dating back to 1973 is genus *Bacillus* [49]. They are prokaryotic aerobic bacteria which are rod-shaped ranging from 1 to 10 μm [50]. Carbonate precipitation is commonly induced by enzymes that can facilitate mineral formation. Recently, enzymes such as urease have been used to improve the precipitation of biominerals [22,24,25,47,[51], [52], [53], [54], [55]]. The enzymes speed up chemical reactions resulting in a chemical change to the environment which facilitates biomineral formation. A pattern that's observed when the urease enzyme is involved in this reaction is a rise in pH which eventually leads to carbonate precipitation (Fig. 2).

**Table 1:** A summary of bacteria that precipitate calcium carbonate polymorphs using urease aided- MICP.

Microorganisms	Source of origin	Reference
Pseudomonas *calcis*	Soil	[49]
Sporosarcina *pasteurii*	ATCC	[73,74]
Multiple bacteria belonging to Bacillus and Pseudomonas group	Soil	[32]
Myxococcus *xanthus*	Soil	(Rodriguez-Navarro et al., 2007, Gonzalez-Munoz et al., 2010)
Pseudomonas and Acinetobacter Genera	Water	(Zamarreňo et al., 2009a, Zamarreňo et al., 2009b)
Halomonas sp. SR4	Soil	[8]
Thalassospira sp.; Halomonas sp.; Bacillus *pumilus*; Pseudomonas *grimontii*	Soil	[35]
Bacillus *megaterium* SS3 & Bacillus *thuringiensis*	Calcareous cave	[54]
Lysinibacillus *sphaericus* CH5 & Sporosarcina *pasteurii* WJ-2	Heavy metal contaminated mine	[6,7,53]
Bacillus *licheniformis*	Soil	[48]
Multiple bacteria belonging to Bacillus group, Pseudomonas *nitroreducens* szh_asesj15 and Sphingopyxis sp. szh_adharsh	Landfill leachate	[27]
Multiple bacteria belonging to Bacillus group	Heavy metal contaminated mine	[75]

For example, when the urease enzyme is involved, the bacteria breaks down the urea into ammonia and carbamic acid [21].

(1) $$CO{\left(NH_{2}\right)}_{2}+H_{2}O\to NH_{2}COOH+NH_{3}$$

Then the carbamic acid degrades into ammonia and bicarbonate [21].

(2) $$NH_{2}COOH+H_{2}O\to NH_{3}+H_{2}CO_{3}$$

The conversion of ammonia into ammonium releases hydroxide ions [56].

(3) $$NH_{3}+H_{2}O\;\to N{H_{4}}^{+}+OH^{-}$$

Followed by the breakdown of bicarbonate into carbonate and release of hydrogen ions to which react with the hydroxide ions to slightly lower the pH leaving free carbonate ions in solution [21].

(4) $$H_{2}CO_{3}\;\to 2H^{+}+2C{O_{3}}^{2-}$$

Calcium or magnesium can be added during the initial stage of the experiment to achieve the target mineral formation. Equations (5), (6) show that calcium carbonate and magnesium carbonate can be formed [43,57].

(5) $$Ca^{2+}\;+C{O_{3}}^{2-}\;\to CaCO_{3}$$

(6) $$Mg^{2+}\;+C{O_{3}}^{2-}\;\to MgCO_{3}$$

**Fig. 3:**
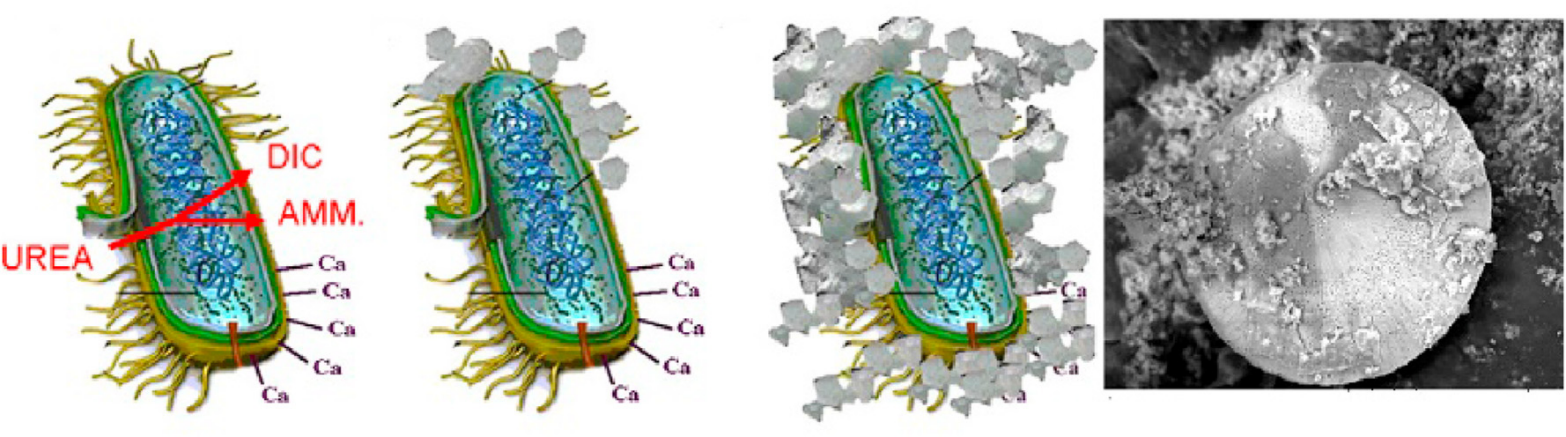
Graphical representation illustrating urea and calcium precipitating calcium carbonate which can be found as imprints on the cell wall of bacteria. Modified from (Muynck et al., 2010a)

During the urea hydrolysis, two major chemical changes can be observed, 1) an abundance of carbonate ions being produced and 2) the rise in pH which happens due to the release of ammonia. Multiple studies have shown that when USB is involved the crystals (biominerals) are attached on the surface of the bacterial cell walls, which serve as nucleation sites (Fig. 3). Since bacterial cell walls are negatively charged, they attract and bind Ca^2+^ ions, which results in multiple bonding locations for Ca^2+^ ions on the surface of the cell wall leading to deposition of biominerals.

## 3 Factors that influence MICP

### 3.1 Temperature

#### 3.1.1 Enzyme activity

Every enzyme related mechanism relies highly on the temperature of its environment. Since bacteria are also sensitive to temperature, optimizing the temperature for MICP is crucial when it comes to its application in the ecosystem. Multiple studies have shown that the ideal temperature for MICP involving USB is 30–35 °C [26,44,58], as the enzyme concentration is superior due to rapid exponential bacterial growth. Nemati and Voordouw [59] demonstrated that a sudden rise from 20 to 50 °C can alter enzymatic activity resulting in 10 times more precipitation of calcium carbonate but overall precipitation was still lower than a consistent 30–35 °C environment. From a general guideline perspective, to precipitate sufficient amount of calcium carbonate using USB, a temperature range of 20–37 °C is optimal [22,60]. [48] reported that temperatures >35 °C result in declining ureolysis when compared with temperatures in the range of 25–35 °C.

#### 3.1.2 Bacterial activity

Temperature was observed and proved to be highly influential on the growth of bacteria that contain the urease enzyme since superior growth leads to higher enzyme release [22,61,62]. From the perspective of bacteria activity, it was determined that a temperature range between 30 °C and 35 °C results in superior bacterial activity leading to higher calcium carbonate precipitation [63]. A comparative study on the effect of low temperature (25 °C) and high temperature (50 °C) towards calcium carbonate precipitation using USB showed that higher temperature yielded superior precipitation [64] but the precipitation was still lower than a consistent 30–35 °C. A study by Zamarreňo et al. [65] showed that isolated bacteria perform better at 25–35 °C, when the temperature gets higher (40 °C) the bacteria die, and when the temperature gets lower (10 °C), they result in low biomineral precipitation. Lower temperature slows or stunts the growth of bacteria resulting in lower urease enzyme activity which affects the overall amount of biomineral precipitation. As reported by Krajewska et al. [66]; a substantial reduction in the enzyme's affinity for the substrate was noted at lower (<15 °C) and higher (<35 °C) temperatures. Apparently, this reduction in affinity at these temperatures can be considered as a resulting of the loss of the active site structure, which is required for the catalysis. Hence, it may be concluded that low temperatures (<20 °C) may not be ideal for the application of MICP.

### 3.2 Substrates

Most of the MICP-USB processes require a nitrogen and calcium source. Since MICP is currently being proposed as an alternative to conventional/traditional remediation techniques, there is also a need for the substrate source to be cost effective. Industrial wastewater has been proposed as an ideal solution due to its high organic matter content [67]. Industrial wastewater usually contains high amounts of calcium as well which could serve as a calcium source for mineral precipitation [[68], [69], [70], [71]]. However, the direct application of wastewater as a potential substrate and calcium source requires further investigation as they contain intractable compounds that could hinder bacterial growth and hence, inhibit biomineral precipitation.

### 3.3 pH

Since ureolysis induces MICP, the starting pH plays a vital role when it comes to carbonate precipitation. Most of the bacteria studied for MICP are heterotrophic facultative bacteria that favor a pH of 7 or above [26]. Controlling the starting pH provides an advantage when it comes to precipitation of metal carbonates. The optimal pH at which ureolysis occurs is constantly under debate since different groups of bacteria have a different pH for releasing the urease enzyme [24,55,[72], [73], [74], [75]]. However, shared technicality among all studies regarding ureolysis is that the release of carbon dioxide and ammonia production increases the pH which helps in maintaining a pH value that's suitable for MICP. Researchers studying metal carbonates and geotechnical engineering found that ureolysis occurs anywhere between 24 and 72 h and starts to decrease after that. This correlates with a spike in pH rise indicating the potential precipitation of carbonates [22,24,25,76,77]. Studies conducted at neutral to high pH (>7) have shown results which indicate superior soil consolidation and a slightly lower performance at acidic to neutral pH (<7). This leads us to believe that most bacteria prefer a slightly acidic to neutral pH as their starting range for metal carbonates and geotechnical studies. Recent studies on urease enzyme producing halophiles and alkaliphiles have shown the ability of these bacteria to survive under high pH conditions and their potential application towards wastewater treatment [17,23,[78], [79], [80]]. Hence, we should acknowledge that pH can influence the transport of heavy metals and bacteria, thus influencing the precipitation and distribution of MICP across contaminated soils.

### 3.4 Bacterial Re-Use after precipitation

It is well documented that for a successful MICP process the bacteria serve as nucleation sites for mineral precipitation and imaging technique have revealed that the bacteria are embedded in carbonate crystals. The challenge that's currently slowing the process of MICP application is due to the fact that bacteria can't be reused after mineral precipitation thus resulting in reduced activity due to mass transfer limitations. A continuous supply of bacteria has to be maintained in order to release the enzyme so that further precipitation can take place for heavy metal carbonation. Despite this minor setback in regards to increased carbonate precipitation, the bacteria embedded in the precipitates would provide a free-biomass effluent, which requires little or no treatment for removing it.

Based on this theory, MICP for heavy metal carbonates has to be a multi batch reactor system, where bacterial growth is maintained and its constantly supplied to the other reactor in which the heavy metal carbonates are precipitated. The first reactor would contain the substrate required for the growth of bacteria, whereas in the second reactor the reagents that are required for carbonate precipitation are supplied. With further research and proper pilot studies, this idea could potentially solve the heavy metal accumulation in wastewater in the field.

## 4 Application of MICP for heavy metals and calcium removal

Over the past few decades, due to urbanisation and excessive anthropogenic activity, heavy metals such as arsenic, cadmium, chromium, lead and zinc are now found at hazardous concentrations (above guidelines) that can pose serious problems to the ecosystem [[81], [82], [83], [84]]. Landfills and wastewater treatment plants are overwhelmed with heavy metal concentrations that are well over the limit of the guidelines issued by the World Health Organization [68,83,85]. Therefore, soil and water have to be constantly monitored for the presence of heavy metals since unsafe levels can cause long term damage to the ecosystem and would need to be removed immediately [70,71,86,87].

When it comes to heavy metal removal from contaminated environments, environmentally unfriendly conventional remediation treatments are still in practice [68,86,88]. These conventional methodologies often remove the heavy metals in insufficient quantities from the environment. On top of that, they are not cost-effective and detectable amounts of heavy metals can still be found in the environment. In addition, high quantities of chemicals and energy are required [68,85,89].

Phytoremediation and bio-sorbents have been long known for their ability to immobilize heavy metals from interacting with the environment [68,90]. Once plants have absorbed heavy metals, they are burned to concentrate the heavy metals in the ash for disposal. This process is repeated in cycles to reduce the concentration of heavy metals in the soil. These treatments still pose a threat where the heavy metal can be released back into the environment if the plant or sorbent decomposes or naturally dies. These techniques can be high cost and take a long time. An ideal situation is where a non-edible, ornamental plant, which can survive the elevated heavy metal concentrations, can be identified and used. This can become a cash crop which can be sold to reduce the cost of the remediation.

In recent times, biomineralization has been introduced to remove heavy metals or convert them from a soluble to an insoluble form from contaminated sites [37]. Multiple studies have shown microorganisms using ureolysis to remove heavy metals (up to 98%) through MICP (Table 2). A recent study by Jalilvand et al. [91] demonstrated that the novel *S. rhizophila* removed 96.25%, 71.3%, and 63.91% of Pb, Cd, and Zn, respectively. In addition, they showed that another novel bacterium *V. boronicumulans* remove 95.93% of Pb, 73.45% of Cd, and 73.81% of Zn. All of these removals were achieved within 72 h of incubation with the bacteria. These percentages of heavy metal carbonates are in par with the popular and efficient *S. pasteurii*.

**Table 2:** **A summary of bacteria that entrap or remove heavy metals**.

Microorganisms	Heavy metal studied	Reference
Enterobacter *cloacae*; Sporosarcina *koreensis*	Lead	[7,77]
Sporosarcina *ginsengisoli*	Arsenic	[9]
Lysinibacillus *sphaericus*; Terrabacter *tumescens*	Cadmium	[6,77]
Kocuria *flava*; Bacillus *subtilis*; Oceanobacillus *indicireducens*; Bacillus *pumilus*	Copper	[92,94]
Sporosarcina sp; Bacillus *subtilis*; Oceanobacillus *indicireducens*; Bacillus *pumilus*	Zinc	[77,94]
Terrabacter *tumescens*	Nickel	[77]
Terrabacter *tumescens*	Cobalt	[77]
Bacillus *subtilis*; Oceanobacillus *indicireducens*; Bacillus *pumilus*	Chromium, Copper and Zinc	[94]
S. *rhizophila* V. *boronicumulans*	Lead, Cadmium and Zinc	[91]

**Fig. 4:**
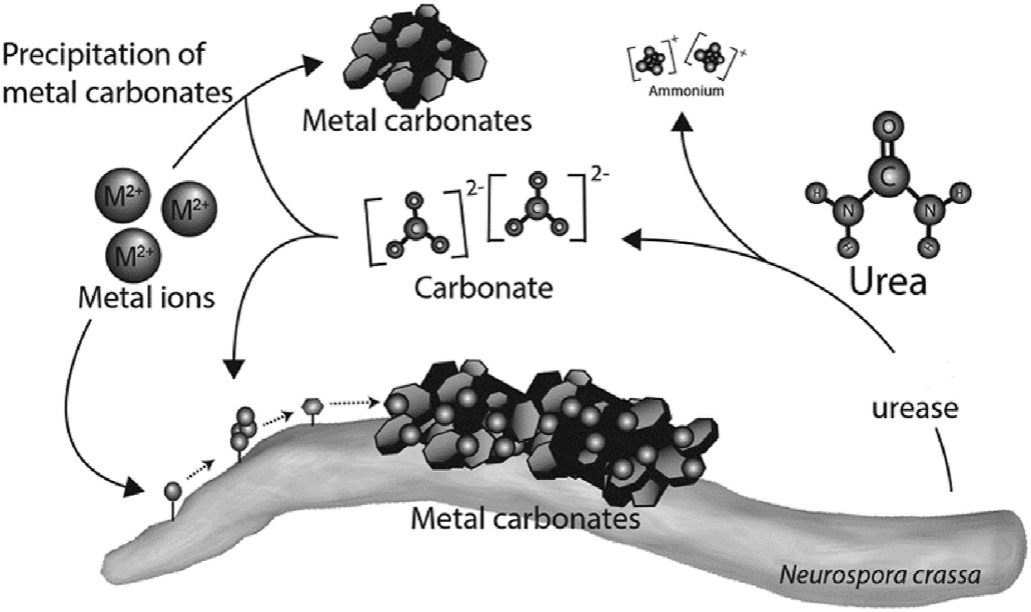
Simplified illustration of metal carbonates formed through MICP. Modified from (Li et al., 2014).

However, bacteria responsible for MICP through ureolysis are not abundant, since they require ideal environmental conditions for releasing the urease enzyme. In many applications, the growth or metabolic activities are limited due to the failure to adapt to the environment. Multiple studies involving the use of bacteria with USB ability isolated from hazardous environments have shown resistance to heavy metals due to the positive effect in heavy metal entrapment [7,9,92,93]. During the process of MICP, heavy metals with divalent ions such as Cd^2+^, Zn^2+^ and Pb^2+^ are substituted for Ca^2+^ which results in the precipitation of heavy metal carbonates thus converting them from a bioavailable to a non-bioavailable form (Equation (7) & Fig. 4) [6,7,94]. Only a few species of bacteria have shown the ability to remove 98% of heavy metals within 48 h of exposure to heavy metals [77]. Indigenous bacteria isolated from heavy metal contaminated soil such as *Bacillus subtilis*, *Oceanobacillus indicireducens* and *Bacillus pumilus* have all shown to be promising in terms of immobilizing heavy metals such as Cr, Cu and Zn in the range of 60–75% in the contaminated soil [94].

(7) $$Pb^{2+}+OH^{-}+HCO_{3}^{-}\to PbCO_{3}+H_{2}O$$

One of the major advantages that MICP has over conventional methodologies is its resistance to redox-insensitive solution making the heavy metal carbonates remain non-toxic, insoluble and non-accessible. The application of MICP in laboratory experiments with an artificial solution spiked with heavy metals has shown potential for heavy metal removal. A 97% copper removal was observed when *Kocuria flava* precipitated carbonates using USB. Application of *Bacillus cereus* through MICP was successful in removing 75% of Cr in contaminated soil [37] and isolation of *Lysinibacillus sphaericus* from South Korean mines has been shown to remove 99.95% of Cd after incubation of 48 h [6]. Li et al. [77] reported a 90% removal of cobalt using MICP-USB isolated from soil. The chemistry behind the efficiency of USB to remove heavy metals such as lead (Pb^2+^), cobalt (Co^2+^) chromium (Cr^2+^), cadmium (Cd^2+^) and copper (Cu^2+^) is due to the fact that these metals share similar divalent ion formation as calcium (Ca^2+^) and also have a large surface area and the abundance of negative ions on the cell surface (Wong, 2015). Lauchnor et al. [95] proposed the application of ureolytic MICP towards the precipitation of heavy metal carbonates as an environmentally friendly engineering application for heavy metal remediation. MICP induced by ureolytic activity has been examined in detail and has also been shown to increase the rate of carbonate precipitation and lead to strontium (Sr^2+^) co-precipitation [96]. MICP by *Bacillus pasteurii* reduced the concentration of Sr^2+^ by 95% within 24 h [97]. Certain scientific studies suggested that strontium can be co-precipitated along with other heavy metals with a divalent cation as long as the metal solution is injected in an optimal way to achieve superior metal carbonation [95,96].

The mineralization of heavy metals within carbonates has additional advantages in that at a high pH, heavy metals are less mobile and if they did reenter solution they will not be transported until the pH of the solution decreased. The presence of solid phase calcium and metal carbonates acts to buffer the pH of the pore solution. A high proportion of the carbonates would need to dissolve in order for the pH to lower sufficiently for the heavy metal ions to become mobile again. Thus, this technique is particularly useful for the long-term immobilization of heavy metal contaminants.

### 4.1 Application of MICP in removing calcium from wastewater

The application of MICP using USB can be applied to treat wastewater which has high amounts of calcium ions. Calcium is one of the hardness causing minerals most commonly released from industries that produce sodium carbonate. High concentrations of calcium in wastewater treatment and reverse osmosis plants show higher scale deposition in pipelines and membranes. The scaling occurs due to the chemical reactions that lead to the formation of multiple calcium products. Since calcium (Ca^2+^) is a vital element in calcium carbonate formation, soluble calcium can be converted into insoluble calcium carbonate and filtered out using MICP. A study conducted by Hammes et al. [32] showed that 90% of Ca^2+^ ions can be removed from industrial wastewaters using MICP. Dong et al. [30] also showed a 91.8% removal of calcium from seawater using MICP.

## 5 Concluding remarks

The body of knowledge of biomineralization has slowly been built to make it a promising sustainable solution in terms of contaminant treatment and entrapment. A wide variety of microorganisms with urease enzymes have been shown to be capable of minimizing the damage caused to the ecosystem through urbanisation and pollution. Multiple microorganisms belonging to the Bacillus group have the most potential for use in MICP. Numerous studies have explored various conventional methodologies for environmental clean-up, but these methods are ineffective and expensive in completely removing the pollutant or contaminant from the environment. Hence, MICP has emerged as an efficient and environmentally friendly method to remediate contaminants such as heavy metals from contaminated environments. Most of the research conducted on heavy metal carbonates are under laboratory conditions or the use of microorganisms that were isolated from heavy metal environments. In addition, there is still a lack of large-scale studies to show its effectiveness and particularly, it remains unclear whether MICP indigenous microorganisms would be capable of removing heavy metals from contaminated sites.

## Declaration of competing interest

The authors declare that they have no known competing financial interests or personal relationships that could have appeared to influence the work reported in this paper..
